# Algorithm-guided goal-directed haemodynamic therapy does not improve renal function after major abdominal surgery compared to good standard clinical care: a prospective randomised trial

**DOI:** 10.1186/s13054-016-1237-1

**Published:** 2016-03-08

**Authors:** Sebastian Schmid, Barbara Kapfer, Markus Heim, Ralph Bogdanski, Aida Anetsberger, Manfred Blobner, Bettina Jungwirth

**Affiliations:** Department of Anaesthesiology, Klinikum rechts der Isar, Technische Universität München, Ismaninger Str. 22, Munich, 81675 Germany

**Keywords:** Goal-directed therapy, Acute kidney injury, Transpulmonary thermodilution, Pulse contour analysis, Major abdominal surgery

## Abstract

**Background:**

Acute kidney injury is a common complication after major surgery. In this study, we investigated whether an algorithm-guided goal-directed haemodynamic therapy (GDT) can improve renal outcome compared to good standard clinical care.

**Methods:**

A total of 180 patients undergoing major abdominal surgery were prospectively and randomly assigned to one of two groups: in the GDT group, patients were treated with a GDT algorithm using transpulmonary thermodilution while standard care was applied to the control patients. Change in creatinine was studied as the primary end point, postoperative complications as well as 1-year mortality as secondary outcomes. Haemodynamics in GDT and control patients were compared calculating goal-achievement rates.

**Results:**

Postoperative change in creatinine (18 ± 39 μmol/l (control) vs. 16 ± 42 μmol/l (GDT); mean difference (95 % confidence interval) 1.6 μmol/l (−10 to 13 μmol/l)) was comparable between the GDT and the control group. Postoperative complications and mortality during hospital stay and after 1 year were not influenced by the use of a GDT algorithm. Achievement rates of haemodynamic goals were not higher in the GDT group compared to the already high (>80 %) rates in the control group. Multivariate regression analysis revealed intraoperative hypotension (MAP < 70 mmHg) and postoperative hypovolaemia (GEDI < 640 ml/m^2^) as risk factors for postoperative renal impairment.

**Conclusions:**

In this study, GDT was not superior to standard clinical care in order to avoid renal failure after major abdominal surgery. The reason for this finding is most likely the high achievement rate of haemodynamic goals in the control group, which cannot be improved by the GDT algorithm.

**Trial registration:**

Clinicaltrials.gov; NCT01035541; registered 17 December 2009.

**Electronic supplementary material:**

The online version of this article (doi:10.1186/s13054-016-1237-1) contains supplementary material, which is available to authorized users.

## Background

Acute kidney injury is a common complication after major non-cardiac surgery and is associated with increased mortality [1–4]. Hypoperfusion and haemodynamic instability resulting in a mismatch of oxygen demand and delivery are discussed in the pathogenesis of postoperative renal impairment, raising the question whether perioperative goal-directed haemodynamic therapy (GDT) might improve postoperative renal outcome [5, 6]. In this context, one meta-analysis focusing on kidney function stated that perioperative haemodynamic optimisation improves renal outcome. However, most of the studies investigated vascular and cardiac surgery patients and used a composite end point of perioperative morbidity in lieu of renal outcome [7]. Therefore, it is not known yet, whether an algorithm-guided GDT is superior to a haemodynamic therapy guided by established clinical standard of care in order to avoid renal failure after non-cardiac surgery.

Any GDT is primarily focusing on haemodynamic stability characterized by achieving defined haemodynamic goals. Therefore, the comparison of an algorithm-guided GDT with standard clinical care requires screening of these haemodynamic goals with an extended GDT monitor in both investigational groups. As just a few studies have recorded achievement rates of haemodynamic goals in both the algorithm-guided GDT as well as the standard clinical care, the conclusion that the use of a GDT algorithm can effectively improve haemodynamic state cannot be drawn yet.

The aim of this study was to investigate if an intra- and postoperative GDT algorithm can improve renal outcomes after major abdominal surgery compared to standard clinical care. Further, this study analysed if the achievement rate of haemodynamic goals is higher in patients treated with a GDT algorithm compared to controls. Finally, other non-renal postoperative complications and mortality were studied up to 1 year after surgery as secondary end points.

## Methods

The study is a prospective randomised trial and was approved by the local ethics committee (Ethikkomission der Fakultät für Medizin der Technischen Universität München; ID: 2538/09) and was registered at the registration site of the US National Institutes of Health (clinicaltrials.gov; Identifier: NCT01035541; principal investigator: Jungwirth Bettina; date of registration 17 December 2009). This prospective, randomised, single-centre study was performed at an University Hospital in Munich, Germany. Patients were included from March 2010 until December 2012 and were followed up to 12 months after surgery, when a telephone interview was performed in order to assess morbidity and mortality. The trial ended after the number of patients determined in the sample size calculation was enrolled.

### Study population

We included patients older than 18 years, American Society of Anesthesiologists (ASA) physical status classification 1–3 undergoing major non-cardiac surgery planned to last at least 3 hours with an expected subsequent intensive care treatment of more than 3 days. Exclusion criteria were need for dialysis and contraindication for an arterial line in the femoral artery. One research team member evaluated patients’ eligibility, informed them in detail about the study and obtained informed consent.

### Anaesthesia

If no contraindications existed, an epidural catheter was inserted before induction of anaesthesia. All patients received general anaesthesia with sufentanil and propofol for induction and sevoflurane for maintenance of anaesthesia. Rocuronium was used as a muscle relaxant. After induction, an 8.5 French central venous catheter was placed in the internal jugular vein and a 5 French thermistor-tipped catheter in the femoral artery. Before surgical incision, an epidural bolus of 10 μg sufentanil and 16 mg ropivacaine was applied. For pain therapy, a continuous epidural infusion of 3 μg sufentanil and 4.8 mg ropivacaine per hour (patient height < 175 cm) or 4 μg sufentanil and 6.4 mg ropivacaine per hour (patient height > 175 cm) was used.

All patients were monitored with a transpulmonary thermodilution monitor (PiCCO2®; PULSION Medical Systems SE, Feldkirchen, Germany). The basal infusion rate during the whole surgery and at the intensive care unit was 100 ml/h of Ringer's acetate (RA) in both groups.

Red packed cells were administered when the haemoglobin level decreased below 8 mg/dl or the patient showed signs of ischaemia like respective alterations in the electrocardiogram. Fresh frozen plasma was given in the presence of coagulopathy assessed using conventional laboratory parameters and ROTEM® (Tem International GmbH, Munich, Germany) diagnostics.

### Randomisation

**Fig. 1:**
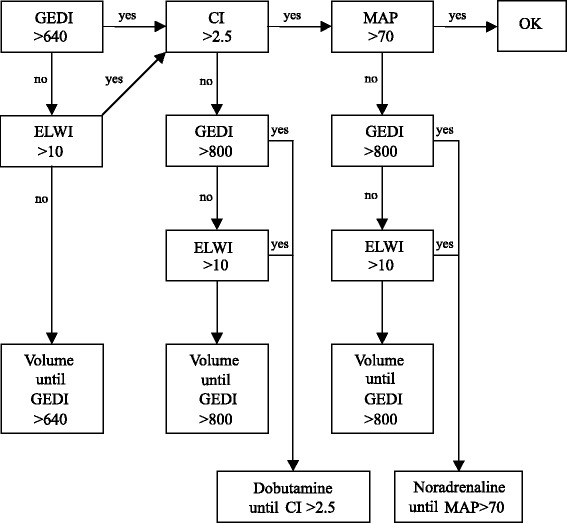
Treatment algorithm in the goal-directed hemodynamic management group, modified according to [8]. *CI* cardiac index, *ELWI* extravascular lung water index, *GEDI* global end-diastolic index, *MAP* mean arterial pressure

Patients were randomly allocated to one of two groups in a 1:1 ratio using a computer-generated list: GDT or control. In the GDT group, the patients’ haemodynamic conditions were treated according to an established algorithm, which is an adaption of the one used by Goepfert in patients undergoing cardiac surgery (Fig. 1) [8]. In the control group, haemodynamics were managed using the standard care of our hospital. Prior to anaesthesia induction a study team member assessed the randomisation list.

### Intraoperative management – GDT group

A resident and an attending anaesthesiologist performed anaesthesia in patients of the GDT group with exclusion of haemodynamic therapy. A member of the research team performed haemodynamic therapy according to the algorithm using the PiCCO® monitor. In detail, transpulmonary thermodilution measurements were carried out every 30 minutes during anaesthesia [8]. Global end-diastolic index (GEDI), mean arterial pressure (MAP) as well as cardiac index (CI) were used as the main objectives. In case the algorithm recommended a fluid bolus, 500 ml of hydroxyethyl starch (HES) or RA were infused within 15 minutes followed by another thermodilution to confirm that goals were achieved. When the study was conducted in our hospital, standard clinical protocol for fluid resuscitation allowed the use of HES 130/0.4 during surgery and during stay in the intensive care unit (ICU) with a maximum dose of 50 ml/kg bodyweight per day. The attending anaesthesiologist/intensivist decided according to personal preferences whether to use HES or RA for fluid resuscitation. In order to consider a potential negative effect of HES on renal function, we recorded the total amount of HES used in our patients. Norepinephrine was used as a vasopressor, dobutamine as an inotrope.

### Intraoperative management – control group

A resident and an attending anaesthesiologist performed anaesthesia in patients of the control group including haemodynamic therapy according to the standard care of our hospital. Haemodynamic monitoring incorporates invasive blood pressure monitoring, which was not able to automatically calculate advanced haemodynamic parameters like pulse pressure variation or systolic pressure variation. Fluids and catecholamines were administered at the attending anaesthetist’s discretion. In addition, a member of the research team implemented the PiCCO® monitor and performed transpulmonary thermodilution after induction and then every 30 minutes but did not communicate the results of the pulse contour or thermodilution measurements. The PICCO® monitor was additionally covered during the whole study period.

### Management in the intensive care unit

In both groups, the monitoring with transpulmonary thermodilution was continued in the intensive care unit until 72 hours after surgery and was calibrated every 8 hours. Again, haemodynamic management in patients of the GDT group was performed according to the algorithm while haemodynamic therapy in patients of the control group was at the intensivist’s discretion. In the control group, the PiCCO® monitor was covered and a person other than the attending caregiver carried out the thermodilution.

### Outcomes

The primary end point was the maximum change in serum creatinine and in creatinine clearance within 7 days after the operation. Therefore, blood samples were taken daily during the ICU stay and at least once on the normal ward. Creatinine clearance was calculated using age and gender according to the modification of diet in renal disease (MDRD) formula.

As pre-specified secondary outcome measures, we assessed the stage of acute postoperative renal failure using the risk, injury, failure, loss of kidney function and end-stage renal disease (RIFLE) criteria during the ICU stay [9]. We analysed creatinine, glomerular filtration rate (GFR) and hourly urine output to determine RIFLE criteria. In addition, we performed an exploratory analysis using these parameters in order to determine the incidence of acute kidney injury according to the Kidney Disease; Improving Global Outcome (KDIGO) definition, a new definition for acute kidney injury updated in 2012 after commencement of our study [6]. After discharge from the ICU the need for dialysis was recorded. Further outcome parameters like surgical re-intervention, respiratory (pulmonary oedema, pleural infection, re-intubation and prolonged ventilation) and cardiocirculatory (myocardial infarction and prolonged hypotension) complications, sepsis and postoperative mortality were assessed during the hospital stay. The incidence of delirium in the ICU was determined with the confusion assessment method (CAM). Acute physiology and chronic health evaluation II (APACHE II) and sequential organ failure assessment (SOFA) scores were evaluated every day during the ICU stay.

### Effect of goal-directed therapy

In order to compare the haemodynamics of GDT and control patients we analysed the achievement rates of haemodynamic goals. For this purpose, all haemodynamic data were saved on the PiCCO® monitor every 12 seconds using the integrated data recording system. In addition, the results of the transpulmonary thermodilution were documented in a case report form. Due to breakdowns of the internal recording system, especially during the early phase of the study, continuous data of 13 patients (seven GDT, six control) are not available. In these patients only the results of the transpulmonary thermodilution have been analysed. The goal-achievement rate was calculated dividing the number of measurements the parameter was within the target range of the algorithm by the total number of measurements.

### One-year follow-up

One year after the operation patients were contacted via telephone. A questionnaire investigating the patients’ state of health (Additional file 1) and a 12-item short-form health survey (SF-12) telephone questionnaire were completed with the patient. In cases where the patient had died, the next-of-kin was asked about the date and cause of death. Furthermore in cases when we were not able to reach the patient, the hospital record was reviewed for information about health status and survival during the last year. We introduced the 1-year follow-up after 17 patients (ten GDT, seven control) had already been included. As these patients had not been informed about the 1-year interview, their data are missing.

### Statistical analysis

Calculation of sample size was based on data from patients who underwent major surgery fulfilling the inclusion criteria in the months March and July 2009 in our hospital. Expecting a reduction of maximum creatinine change by 40 % (80 % power, *p* < 0.05 at two-sided error) a sample size of 90 patients per group was calculated.

In a confirmatory approach, primary as well as secondary outcomes were compared between GDT and standard treatment. Mean or median difference and their 95 % confidence interval (CI) were evaluated with independent *t* test or Mann-Whitney *U* test according to their distribution. Dichotomic parameters were compared with odds ratio and 95 % CI by generalised linear modelling of a binary logistic regression model. Significance level was *p* < 0.05.

In an observatory approach, the effects of further risk factors as well as the achievement rates of haemodynamic objectives on postoperative renal outcome were analysed with a multivariate linear regression model using the absolute decrease of the creatinine clearance as dependent variable. We tested the independent factors, type of fluid, age, sex, body mass index, infusion rate of noradrenaline and dobutamine and the ratio within the target range of mean arterial pressure, cardiac index, global end-diastolic index and extravascular lung water index during the operation and the ICU observation period.

Calculations were done with IBM SPSS Statistics® (Version 21.0; IBM Corp., Armonk, NY, USA). Metric data are expressed as mean and standard deviation (SD) or median and range (minimum to maximum) as appropriate, categorical data as number and percentage.

## Results

### Patient characteristics, anaesthesia, and haemodynamic therapy

**Fig. 2:**
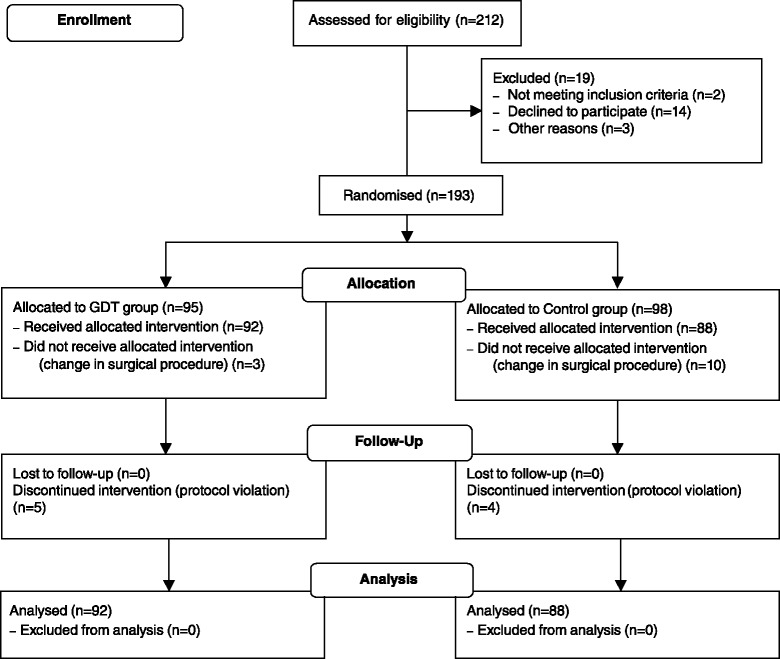
CONSORT flow diagram. *GDT* goal-directed haemodynamic therapy

We screened 212 patients for eligibility. A total of 193 patients were randomised and 180 analysed. Thirteen patients were excluded from the analysis, as they did not receive the allocated intervention, that means surgery was terminated due to unexpected findings during the operation (for example metastases, peritoneal carcinosis). Ninety-two patients in the GDT group received the allocated intervention and 88 in the control group. Although in nine patients (five GDT, four control) protocol violations were observed, we decided to include these patients in our analysis (Fig. 2). Baseline characteristics showed no difference between the two groups regarding preoperative serum creatinine or creatinine clearance or pre-medical condition (Table 1). Due to contraindications five patients did not receive epidural anaesthesia (three GDT, two control).

**Table 1:** Baseline characteristics

	Control	GDT
Age (year)	65 ± 11	67 ± 12
Body mass index (kg/m^2^)	21 ± 4	22 ± 5
Gender		
Male	70 (80 %)	68 (74 %)
Female	18 (20 %)	24 (26 %)
Preoperative renal function		
Creatinine (μmol/l)	79 ± 23	81 ± 22
Creatinine clearance (ml/min/1.73 m^2^)	87 ± 26	83 ± 25
Type of surgery		
Whipple	14 (16 %)	16 (18 %)
Oesophageal	65 (74 %)	71 (77 %)
Pancreatectomy	5 (6 %)	3 (3 %)
Other	4 (4 %)	2 (2 %)
Pre-medical condition		
Diabetes	15 (17 %)	21 (23 %)
Hypertension	43 (48 %)	58 (63 %)
Chronic renal failure	3 (3 %)	6 (7 %)
Coronary artery disease	12 (13 %)	19 (21 %)
Heart failure	4 (4 %)	7 (8 %)
Arrhythmia	6 (7 %)	12 (13 %)
Valvular disorder	3 (3 %)	3 (3 %)
Haemodynamics after induction		
GEDI (ml/m^2^)	742 ± 164	715 ± 141
MAP (mmHg)	79 ± 17	78 ± 16
CI (ml/min/m^2^)	2.4 ± 0.5	2.4 ± 0.6
ELWI (ml/kg)	8 ± 2	8 ± 2

Categorical variables are given as number and percentage, continuous variables as mean ± standard deviation

*GDT* goal-directed haemodynamic therapy, *GEDI* global end-diastolic index, *MAP* mean arterial pressure, *CI* cardiac index, *ELWI* extravascular lung water index

The total amount of RA and HES infused did not differ between the GDT and control group. More dobutamine was used in the GDT group during surgery compared to the control group. The amount of vasopressor was not different between groups (Table 2). The overall transfusion rate was low with 0.5 red packed cells and 0.3 fresh frozen plasma units per patient and was not different between groups. In total only 41 patients received red packed cells and 14 patients fresh frozen plasma units.

**Table 2:** Fluids and catecholamines administered. Comparison between intervention groups

		Control	GDT	Mean diff. (95 % CI)	*p* value
Fluids					
RA (ml)	OP	2316 ± 1800	2529 ± 2169	202 (−210 to 614)	0.34
	ICU	8052 ± 3277	8597 ± 3147	557 (−386 to 1499)	0.25
HES (ml)	OP	847 ± 1045	801 ± 1080	−50 (−237 to 138)	0.60
	ICU	701 ± 1188	514 ± 1083	−199 (−489 to 88)	0.17
Catecholamines					
Noradrenaline (μg/kg/min)	OP	0.03 ± 0.03	0.03 ± 0.06	0.00 (−0.02 to 0.01)	0.71
	ICU	0.01 ± 0.04	0.03 ± 0.08	0.02 (−0.01 to 0.03)	0.14
Dobutamine (μg/kg/min)	OP	0.00 ± 0.03	0.74 ± 2.61	0.73 (0.18 to 1.29)	0.01
	ICU	0.13 ± 0.96	0.39 ± 1.56	0.26 (−0.13 to 0.64)	0.19

Variables are given as mean ± standard deviation

*GDT* goal-directed haemodynamic therapy, *diff*. difference, *CI* confidence interval, *RA* Ringer’s acetate, *HES* hydroxyethyl starch, *OP* operation, *ICU* intensive care unit (the first 72 hours)

### Confirmatory analyses

The change in creatinine and creatinine clearance within the first 7 postoperative days did not differ between the GDT and control group. RIFLE criteria determined during ICU stay as well as the need for dialysis after ICU were not different between the two groups. The exploratory analysis according to the KDIGO definition of acute kidney injury did not detect any differences between the two groups. No patient developed end-stage renal disease.

The incidence of all postoperative complications as well as mortality was not different between the two study groups (Table 3). There was also no difference between the control and GDT groups regarding APACHE II (6.7 ± 2.8 vs. 6.9 ± 1.4) and SOFA (1.1 ± 1.7 vs. 1.4 ± 1.7) score.

**Table 3:** Primary as well as short- and long-term secondary outcome parameters

	Control	GDT	Mean diff. (95 % CI)	Odds ratio (95 % CI)	*p* value
Primary renal outcomes					
Change in creatinine (μmol/l)	18 ± 39	16 ± 42	1.6 (−10 to 13)		0.788
Change in creatinine clearance (ml/min/1.73 m^2^)	−12 ± 24	−10 ± 24	−2 (−9 to 5)		0.566
Secondary outcomes (hospital stay)					
Incidence of acute kidney injury according to RIFLE (first line: all patients with RIFLE ≥ 1)	45/88 (51 %)	54/92 (59 %)		0.73 (0.41 to 1.33)	0.31
	R: 29/88	R: 30/92			
	I: 14/88	I: 22/92			
	F: 2/88	F: 2/92			
Incidence of acute kidney injury according to KDIGO (first line: all patients with AKI ≥ 1) (Exploratory endpoint)	46/88 (52 %)	53/92 (58 %)		0.81 (0.45 to 1.45)	0.47
	1: 34/88	1: 30/92			
	2: 10/88	2: 21/92			
	3: 2/88	3: 2/92			
Need for dialysis after RIFLE observation period	4/88 (5 %)	5/92 (5 %)		0.83 (0.21 to 3.23)	0.78
Incidence of ≥1 surgical re-intervention	19/88 (22 %)	24/92 (26 %)		0.78 (0.39 to 1.56)	0.48
Incidence of ≥1 postoperative respiratory complication^a^	28/88 (32 %)	28/92 (30 %)		1.07 (0.57 to 2.01)	0.84
Incidence of ≥1 postoperative cardiocirculatory complication^b^	2/88 (2 %)	6/92 (7 %)		0.33 (0.07 to 1.70)	0.19
Incidence of postoperative sepsis	7/88 (8 %)	7/92 (8 %)		1.05 (0.35 to 3.19)	0.93
Incidence of postoperative delirium	12/70 (17 %)	18/75 (24 %)		0.65 (0.29 to 1.48)	0.31
In-hospital mortality	4/88 (5 %)	4/92 (4 %)		0.79 (0.28 to 2.21)	0.65
Secondary outcome (one year)					
Incidence of hospital re-admission	36/65 (55 %)	28/65 (43 %)		1.64 (0.82 to 3.28)	0.16
Incidence of re-admission to ICU	11/64 (17 %)	11/65 (17 %)		1.02 (0.41 to 2.55)	0.97
Incidence of surgical re-intervention	28/67 (42 %)	20/66 (30 %)		1.65 (0.08 to 3.38)	0.17
Incidence of new need for dialysis after discharge	1/64 (2 %)	2/63 (3 %)		0.48 (0.04 to 5.48)	0.56
Incidence of myocardial infarction	0/64 (0 %)	3/65 (5 %)		n.a.	0.24^c^
Incidence of stroke	1/64 (2 %)	0/63 (0 %)		n.a.	1.00^c^
SF-12 Quality of life physical sum score	45.3 ± 9.1	45.0 ± 11.1	0.25 (−4.9 to 5.4)		0.92
SF-12 Quality of life mental sum score	50.6 ± 10.4	50.9 ± 10.3	−0.27 (−5.5 to 5.0)		0.92
One-year overall mortality	16/77 (21 %)	22/77 (29 %)		0.66 (0.31 to 1.37)	0.26

Variables of primary renal outcome and SF-12 are given as mean ± standard deviation and variables of secondary outcome as number of patients showing the complication (n) in relation to the number of treated patients within the respective group (N) and incidence (%)

*GDT* goal-directed haemodynamic therapy, *diff*. difference, *CI* confidence interval, *RIFLE* risk, injury, failure, loss, end-stage kidney disease, *R* risk, *I* injury, *F* failure, *KDIGO* Kidney Disease: Improving Global Outcome, *AKI* acute kidney injury, *ICU* intensive care unit, *n.a.* not applicable, SF-12 12-item short-form health survey

^a^Pulmonary edema, pleural infection, re-intubation and prolonged ventilation

^b^Myocardial infarction and prolonged hypotension

^c^Fisher’s exact test

One year after surgery we tried to contact 163 of the 180 patients. Seventeen were not contacted, as their informed consent for the telephone interview was missing due to the later amendment to the ethics committee as mentioned in the Methods section. For determination of 1-year mortality (including in-hospital mortality), we combined the information on the patients’ electronic record with that of the telephone interviews. Therefore, data on 1-year mortality was available for 154 of the 163 patients investigated (94 %) and did not differ between the two groups (Table 3). All other long-term outcome parameters did not differ between the control and GDT groups as well (Table 3).

Implementation of a GDT algorithm did not result in higher rates of achieving haemodynamic objectives when compared to control patients. In contrast, MAP was more often above 70 mmHg in control patients as compared to GDT patients. The median achievement rates of CI were high and comparable between patients of the GDT and the control group. The minimum of the achievement rate was low for CI in both groups so that some patients did not reach the designated CI > 2.5 l/min/m^2^. Furthermore all haemodynamic parameters were more often within target ranges during ICU stay when compared to the operation period (Table 4).

**Table 4:** Comparison of achievement rates between intervention groups and between surgery and intensive care unit

		Control	GDT	Control vs. GDT		OP vs. ICU	
				Median diff. (95 % CI)	*p* value	Median diff. (95 % CI)	*p* value
Pulse contour data							
MAP > 70 mmHg	OP	0.85	0.77	−0.06	0.010	0.08	< 0.001
		(0.33 – 1.0)	(0.33 – 0.99)	(−0.11 to −0.02)		(0.05 to 0.12)	
	ICU	0.91	0.90	0.00	0.74		
		(0.10 – 1.0)	(0.29 – 1.0)	(−0.03 to 0.02)			
CI > 2.5 l/min/m^2^	OP	0.82	0.75	−0.04	0.20	0.23	< 0.001
		(0.01 – 1.0)	(0.06 – 1.0)	(−0.10 to 0.02)		(0.18 to 0.27)	
	ICU	0.99	0.97	0.00	0.12		
		(0.72 - 1.0)	(0.59 – 1.0)	(−0.02 to 0.00)			
Thermodilution data							
CI > 2.5 l/min/m^2^	OP	0.79	0.71	−0.04	0.22	0.27	< 0.001
		(0.0 – 1.0)	(0.0 – 1.0)	(−0.13 to 0.00)		(0.23 to 0.31)	
	ICU	1.0	1.0	0.00	0.18		
		(0.56 – 1.0)	(0.50 – 1.0)	(0.00 to 0.00)			
GEDI > 640 ml/m^2^	OP	0.90	1.0	0.00	0.70	0.22	0.001
		(0.0 – 1.0)	(0.0 – 1.0)	(0.00 to 0.00)		(0.00 to 0.71)	
	ICU	1.0	1.0	0.00	0.18		
		(0.0 – 1.0)	(0.0 – 1.0)	(0.00 to 0.00)			
ELWI ≤ 10 ml/kg	OP	1.00	1.00	0.00	0.06	0.00	0.012
		(0.0 – 1.0)	(0.0 – 1.0)	(0.00 to 0.00)		(0.00 to 0.00)	
	ICU	1.00	1.00	0.00	0.10		
		(0.13 – 1.0)	(0.0 – 1.0)	(0.00 to 0.00)			

Variables are given as median of the ratio the respective parameter was within the target range relating to the complete observation period and range (minimum to maximum); continuous data is haemodynamic data saved every 12 seconds with the integrated recording system. Pulse contour data is haemodynamic data acquired whenever thermodilution was performed

*GDT* goal-directed haemodynamic therapy, *OP* operation, *ICU* intensive care unit, *diff.* difference, *CI* confidence interval, *MAP* mean arterial pressure; *CI* cardiac index; *GEDI* global end-diastolic index; *ELWI* extravascular lung water index

### Exploratory analysis

Multivariate regression analysis showed an impact of body mass index and preoperative creatinine clearance on postoperative change in creatinine clearance. Further, the achievement rate of MAP above 70 mmHg during operation, and the achievement rate of GEDI above 640 ml/m^2^ during ICU stay influenced the postoperative renal outcome. Last but not least, the multivariate regression analysis showed a negative impact of HES on the postoperative creatinine clearance. The achievement rates of all other haemodynamic objectives and the infusion rate of catecholamines demonstrated no impact on renal outcome (Table 5).

**Table 5:** Multivariate linear regression analysis of factors influencing change of creatinine clearance

	Means	Regression coefficient (95 % CI)		*p* value
Fluid [= HES]	48 %	−8.2	(−15.7 to −0.7)	0.033
Sex [= male]	77 %	1.7	(−7.0 to 10.4)	0.697
Age (year)	66 (64 to 67)	−0.4	(−0.8 to 0.0)	0.054
BMI (kg/m^2^)	22 (21 to 22)	−1.8	(−2.7 to −0.8)	< 0.001
Preoperative creatinine clearance (ml/min/1.73 m^2^)	87 (83 to 91)	−0.4	(−0.6 to −0.2)	< 0.001
MAP > 70 mmHg (achievement rate during whole operation period)	0.77 (0.74 to 0.80)	27.9	(5.9 to 49.8)	0.013
MAP > 70 mmHg (achievement rate during whole ICU observation)	0.85 (0.83 to 0.88)	19	(−2.4 to 41)	0.081
CI > 2.5 l/min/m^2^ (achievement rate during whole operation period)	0.70 (0.66 to 0.74)	−5.8	(−22 to 10)	0.477
CI > 2.5 l/min/m^2^ (achievement rate during whole ICU observation)	0.95 (0.94 to 0.96)	16.7	(−36.6 to 70.1)	0.536
Noradrenaline infusion rate^a^ during operation (μg/kg/min)	0.03 (0.02 to 0.04)	−42	(−111 to 26)	0.222
Noradrenaline infusion rate^a^ during ICU (μg/kg/min)	0.02 (0.01 to 0.03)	−60	(−126 to 5.6)	0.073
Dobutamine infusion rate^a^ during operation (μg/kg/min)	0.40 (0.07 to 0.73)	1.4	(−0.9 to 3.8)	0.227
Dobutamine infusion rate^a^ during ICU (μg/kg/min)	0.17 (0.02 to 0.32)	−1.7	(−5.6 to 2.2)	0.389
GEDI > 640 ml/m^2^ (achievement rate during whole operation period)	0.74 (0.69 to 0.79)	−9.2	(−26.9 to 8.4)	0.302
GEDI > 640 ml/m^2^ (achievement rate during whole ICU observation)	0.80 (0.76 to 0.85)	20	(2.3 to 37)	0.027
ELWI < 11 ml/kg (achievement rate during whole operation period)	0.89 (0.86 to 0.93)	15	(−1.8 to 31)	0.079
ELWI < 11 ml/kg (achievement rate during whole ICU observation)	0.93 (0.90 to 0.96)	−3.2	(−24 to 18)	0.764

Means are given as percentage of all patients, mean of all patients (95 % confidence interval) or mean of the ratio the respective parameter was within the target range relating to the complete observation period of all patients (95 % confidence interval)

*CI* confidence interval, *HES* hydroxyethyl starch, *BMI* body mass index, *MAP* mean arterial pressure, *ICU* intensive care unit, *CI* cardiac index, *GEDI* global end-diastolic index, *ELWI* extravascular lung water index

^a^Mean individual infusion rate

In the operating theatre the achievement rate of MAP, GEDI and CI was significantly better in patients who were treated with HES compared to RA. Median difference (95 % confidence interval) and *p* value for HES compared to RA: MAP > 70 mmHg: 0.06 (0.02 to 0.11) *p* = 0.006; CI > 2.5 l/min/m^2^: 0.10 (0.03 to 0.19) *p* = 0.002; GEDI > 640 ml/m^2^: 0.11 (0.00 to 0.23) *p* < 0.0019. This effect was not evident at the intensive care unit.

## Discussion

In our study an algorithm-guided GDT was not accompanied with a reduced risk of postoperative renal failure or other short- and long-term complications compared to good standard clinical care. This is most likely due to the unexpected high achievement rate of haemodynamic goals in control patients with no further improvement in patients in whom the GDT algorithm was applied. Using a multivariate analysis, short-term postoperative renal outcome was influenced by body mass index and preoperative creatinine clearance as immutable factors as well as by intraoperative hypotension, postoperative hypovolaemia and the use of HES as controllable factors.

The question of whether GDT improves postoperative outcome is still under debate, just as the discussion about the best monitor [10–15]. Studies providing controversial results used heterogeneous haemodynamic monitors with different algorithms, starting haemodynamic therapy at different times for various durations. Our study focused on renal impairment, which could occur during or after major surgery. Therefore, it was of importance to study the whole perioperative period of intensive observation that is intraoperative and in the ICU [16, 17]. This long observation period with different ventilation modalities from controlled ventilation during the operation to spontaneous breathing in the ICU requires a haemodynamic monitor, which enables the assessment of preload independent of the mode of ventilation. GEDI as a volumetric parameter has been shown to be valid during different ventilation modes and in the presence of arrhythmia [18]. Next to preload, cardiac output and perfusion pressure are discussed as important parameters in order to avoid postoperative complications [12, 19]. Therefore, we employed the PiCCO® monitor with Goepfert’s algorithm using GEDI, CI and MAP as the main objectives [8].

Several studies have shown that algorithm-guided GDT can improve postoperative outcomes. The underlying mechanisms are assumed to be better haemodynamic stability compared to standard clinical care [14]. However, haemodynamic stability characterized by better achievement of haemodynamic goals was rarely measured in standard clinical care patients. The few studies reporting these achievement rates showed very low rates in control patients (19 % and 60 %) [20, 21]. In contrast, our study revealed achievement rates of GEDI, CI and MAP in control patients with 80 % and more as very high, which might be one reason why the algorithm was not able to raise the achievement rates in the GDT group. Another reason could be the algorithm itself: in the GDT group no preventive measures were undertaken when the MAP was decreasing slowly, instead hypotension was not treated until the MAP was below 70 mmHg. In addition, for the right treatment a thermodilution was performed as a time-consuming measurement. In the control group, the anaesthesiologist had no strict protocol to treat or prevent hypotension resulting in better rates of achieving the goal for MAP.

The median of the achievement rates was very high (Table 3), while the range (minimum to maximum) was wide, raising the question, whether a low achievement rate of haemodynamic goals is associated with an increased risk for renal impairment. Therefore, post hoc multivariate regression analysis was performed and demonstrated that avoiding intraoperative hypotension and postoperative hypovolaemia is vital for short-term renal function. In other words, the mere application of an algorithm does not improve renal outcome as long as it does not sufficiently avoid haemodynamic instability.

To date, the debate about the safety concerns of HES is still ongoing culminating in recommendations that the use of HES should be suspended in critically ill patients [22, 23]. These recommendations based on several clinical trials and meta-analyses showing negative effects of HES on renal function and mortality in ICU patients [24–27]. In contrast, some studies were not able to confirm these detrimental effects for the perioperative setting [28–31]. In our study, post hoc multivariate regression analysis has shown that the use of HES presents an independent risk factor for postoperative renal impairment, although the haemodynamic goals were better achieved with HES compared to RA.

Results from controlled clinical trials can be transferred to clinical routine with limitations only. Just the presence of a covered PiCCO® monitor could have influenced the attending anaesthesiologist’s haemodynamic management. Another limitation could be the patient population: we also included healthy individuals undergoing major surgery. Surprisingly, our patients showed a higher incidence of renal impairment (55 %) according to RIFLE criteria compared to other studies [1, 2]. This could be in part due to the fact that we analysed not only serum creatinine and creatinine clearance but also hourly urine output on the ICU to determine the highest RIFLE level, which leads to a more accurate estimation of renal impairment [32]. The incidence of renal impairment without analysis of urine output in our patient population is 27 % and therefore comparable to other studies reporting postoperative acute kidney injury [1]. These incidences could be confirmed in an exploratory analysis when the newer KDIGO definition of acute kidney injury was applied (incidence 55 % with and 24 % without analysis of urine output).

## Conclusions

In conclusion, an algorithm-guided GDT using PiCCO2® monitoring was not able to reduce renal failure after major abdominal surgery in this study. This finding is most likely owing to the already high achievement rates of haemodynamic goals in the standard clinical care group. Exploratory analysis revealed intraoperative hypotension (MAP < 70 mmHg), postoperative hypovolaemia (GEDI < 640 ml/m^2^) and the use of HES as modifiable risk factors for short-term postoperative renal failure.

## Key messages

- Algorithm-guided GDT did not further improve haemodynamic stability compared to good standard clinical care in our study.
- Therefore algorithm-guided GDT does not reduce incidence of renal failure after major non-cardiac surgery.
- Risk factors for postoperative renal failure are intraoperative hypotension, postoperative hypovolaemia and the use of HES.

## Additional file

Additional file 1: Telephone questionnaire. Telephone questionnaire of the 1-year follow-up. (PDF 76 kb)

## Abbreviations

APACHE II: Acute physiology and chronic health evaluation II
CAM: Confusion assessment method
CI: Cardiac index
GDT: Goal-directed haemodynamic therapy
GEDI: Global end-diastolic index
GFR: Glomerular filtration rate
HES: Hydroxyethyl starch
ICU: Intensive care unit
KDIGO: Kidney Disease: Improving Global Outcome
MAP: Mean arterial pressure
MDRD: Modification of diet in renal disease
RA: Ringer's acetate
RIFLE: Risk, injury, failure, loss of kidney function and end-stage renal disease
SF-12: 12-Item short-form health survey
SOFA: Sequential organ failure assessment

## References

[1] Bihorac A, Yavas S, Subbiah S, Hobson CE, Schold JD, Gabrielli A (2009). Long-term risk of mortality and acute kidney injury during hospitalization after major surgery. Ann Surg.

[2] Hobson CE, Yavas S, Segal MS, Schold JD, Tribble CG, Layon AJ (2009). Acute kidney injury is associated with increased long-term mortality after cardiothoracic surgery. Circulation.

[3] Biteker M, Dayan A, Tekkesin AI, Can MM, Tayci I, Ilhan E (2014). Incidence, risk factors, and outcomes of perioperative acute kidney injury in noncardiac and nonvascular surgery. Am J Surg..

[4] O’Connor ME, Kirwan CJ, Pearse RM, Prowle JR. Incidence and associations of acute kidney injury after major abdominal surgery. Intensive Care Med. 2015. Epub ahead of print. 10.1007/s00134-015-4157-7 26602784

[5] Tang IY, Murray PT (2004). Prevention of perioperative acute renal failure: what works?. Best Pract Res Clin Anaesthesiol..

[6] Kidney Disease: Improving Global Outcomes (KDIGO) Acute Kidney Injury Work Group: KDIGO clinical practice guideline for acute kidney injury. Kidney Int Suppl. 2012;2:1–138.

[7] Brienza N, Giglio MT, Marucci M, Fiore T (2009). Does perioperative hemodynamic optimization protect renal function in surgical patients? A meta-analytic study. Crit Care Med..

[8] Goepfert MS, Reuter DA, Akyol D, Lamm P, Kilger E, Goetz AE (2007). Goal-directed fluid management reduces vasopressor and catecholamine use in cardiac surgery patients. Intensive Care Med..

[9] Bellomo R, Ronco C, Kellum JA, Mehta RL, Palevsky P (2004). Acute renal failure - definition, outcome measures, animal models, fluid therapy and information technology needs: the Second International Consensus Conference of the Acute Dialysis Quality Initiative (ADQI) Group. Crit Care..

[10] Bartha E, Davidson T, Brodtkorb TH, Carlsson P, Kalman S (2013). Value of information: interim analysis of a randomized, controlled trial of goal-directed hemodynamic treatment for aged patients. Trials..

[11] Benes J, Chytra I, Altmann P, Hluchy M, Kasal E, Svitak R (2010). Intraoperative fluid optimization using stroke volume variation in high risk surgical patients: results of prospective randomized study. Crit Care..

[12] Donati A, Loggi S, Preiser JC, Orsetti G, Munch C, Gabbanelli V (2007). Goal-directed intraoperative therapy reduces morbidity and length of hospital stay in high-risk surgical patients. Chest..

[13] Goepfert MS, Richter HP, Zu Eulenburg C, Gruetzmacher J, Rafflenbeul E, Roeher K (2013). Individually optimized hemodynamic therapy reduces complications and length of stay in the intensive care unit: a prospective, randomized controlled trial. Anesthesiology..

[14] Hamilton MA, Cecconi M, Rhodes A (2011). A systematic review and meta-analysis on the use of preemptive hemodynamic intervention to improve postoperative outcomes in moderate and high-risk surgical patients. Anesth Analg..

[15] Salzwedel C, Puig J, Carstens A, Bein B, Molnar Z, Kiss K (2013). Perioperative goal-directed hemodynamic therapy based on radial arterial pulse pressure variation and continuous cardiac index trending reduces postoperative complications after major abdominal surgery: a multi-center, prospective, randomized study. Crit Care..

[16] Bartels K, Karhausen J, Clambey ET, Grenz A, Eltzschig HK (2013). Perioperative organ injury. Anesthesiology..

[17] Bellomo R, Auriemma S, Fabbri A, D'Onofrio A, Katz N, McCullough PA (2008). The pathophysiology of cardiac surgery-associated acute kidney injury (CSA-AKI). Int J Artif Organs..

[18] Michard F, Alaya S, Zarka V, Bahloul M, Richard C, Teboul JL (2003). Global end-diastolic volume as an indicator of cardiac preload in patients with septic shock. Chest..

[19] Berlauk JF, Abrams JH, Gilmour IJ, O'Connor SR, Knighton DR, Cerra FB (1991). Preoperative optimization of cardiovascular hemodynamics improves outcome in peripheral vascular surgery. A prospective, randomized clinical trial. Ann Surg.

[20] Bartha E, Arfwedson C, Imnell A, Fernlund ME, Andersson LE, Kalman S (2013). Randomized controlled trial of goal-directed haemodynamic treatment in patients with proximal femoral fracture. Br J Anaesth..

[21] Lobo SM, Ronchi LS, Oliveira NE, Brandao PG, Froes A, Cunrath GS (2011). Restrictive strategy of intraoperative fluid maintenance during optimization of oxygen delivery decreases major complications after high-risk surgery. Crit Care..

[22] European Medicines Agency. Hydroxyethyl-starch solutions (HES) no longer to be used in patients with sepsis or burn injuries or in critically ill patients. 2013. EMA/809470/2013. Available from: http://www.ema.europa.eu/docs/en_GB/document_library/Referrals_document/Solutions_for_infusion_containing_hydroxyethyl_starch/European_Commission_final_decision/WC500162361.pdf. Accessed 18/09/2015.

[23] Food and Drug Administration. Hydroxyethyl starch solutions: FDA safety communication - boxed warning on increased mortality and severe renal injury and risk of bleeding. 2013. Available from: http://www.fda.gov/Safety/MedWatch/SafetyInformation/SafetyAlertsforHumanMedicalProducts/ucm358349.htm. Accessed 18/09/2015.

[24] Brunkhorst FM, Engel C, Bloos F, Meier-Hellmann A, Ragaller M, Weiler N (2008). Intensive insulin therapy and pentastarch resuscitation in severe sepsis. N Engl J Med..

[25] Myburgh JA, Finfer S, Bellomo R, Billot L, Cass A, Gattas D (2012). Hydroxyethyl starch or saline for fluid resuscitation in intensive care. N Engl J Med..

[26] Perner A, Haase N, Guttormsen AB, Tenhunen J, Klemenzson G, Aneman A (2012). Hydroxyethyl starch 130/0.42 versus Ringer's acetate in severe sepsis. N Engl J Med.

[27] Mutter TC, Ruth CA, Dart AB (2013). Hydroxyethyl starch (HES) versus other fluid therapies: effects on kidney function. Cochrane Database Syst Rev..

[28] Feldheiser A, Pavlova V, Bonomo T, Jones A, Fotopoulou C, Sehouli J (2013). Balanced crystalloid compared with balanced colloid solution using a goal-directed haemodynamic algorithm. Br J Anaesth..

[29] Martin C, Jacob M, Vicaut E, Guidet B, Van Aken H, Kurz A (2013). Effect of waxy maize-derived hydroxyethyl starch 130/0.4 on renal function in surgical patients. Anesthesiology.

[30] Van Der Linden P, James M, Mythen M, Weiskopf RB (2013). Safety of modern starches used during surgery. Anesth Analg..

[31] Yates DR, Davies SJ, Milner HE, Wilson RJ (2014). Crystalloid or colloid for goal-directed fluid therapy in colorectal surgery. Br J Anaesth..

[32] Wlodzimirow KA, Abu-Hanna A, Slabbekoorn M, Chamuleau RA, Schultz MJ, Bouman CS (2012). A comparison of RIFLE with and without urine output criteria for acute kidney injury in critically ill patients. Crit Care..

