# Supplementary material for: Algorithm-guided goal-directed haemodynamic therapy does not improve renal function after major abdominal surgery compared to good standard clinical care: a prospective randomised trial
Source: Crit Care. 2016 Mar 8;20:50. doi: 10.1186/s13054-016-1237-1 (PMC4782303; doi:10.1186/s13054-016-1237-1)
Supplement: Additional file 1: — Telephone questionnaire. Telephone questionnaire of the 1-year follow-up. (PDF 76 kb) [file 13054_2016_1237_MOESM1_ESM.pdf]

One-year follow up

Telephone questionnaire

**Question 1:** After the surgery on … did you have another operation?	
	If yes: When? Where? Kind of Surgery?
	No

**Question 2:** After the surgery on … were you admitted to intensive care unit?	
	If yes: When? Where? Why? How long?
	No

**Question 3:** After the surgery on … were you admitted to hospital?	
	If yes: When? Where? Why? How long?
	No

**Question 4:** Have you been dialysed within the last year?	
	If yes: When? Where? How often?
	No

**Question 5:** Have you had a heart attack within the last year?	
	If yes: When? Where? Therapy?
	No

**Question 6:** Have you had a stroke within the last year?	
	If yes: When? Where? Therapy? Neurologic deficit?
	No

**If patient died within the last year:**	
Date of death:	
Cause of death:	
If death within a hospital:<br>name of the hospital:	
